# Tricking the Brain with Leptin to Limit Post Liposuction and Post Bariatric Surgery Weight Regain?

**DOI:** 10.3390/diseases10040080

**Published:** 2022-10-04

**Authors:** Abdelaziz Ghanemi, Mayumi Yoshioka, Jonny St-Amand

**Affiliations:** 1 Department of Molecular Medicine, Faculty of Medicine, Laval University, Québec, QC G1V 0A6, Canada; 2 Functional Genomics Laboratory, Endocrinology and Nephrology Axis, CHU de Québec-Université Laval Research Center, Québec, QC G1V 4G2, Canada

**Keywords:** obesity, liposuction, bariatric surgery, leptin, weight regain, weight management

## Abstract

Obesity represents a medical challenge for modern therapists. The main difficulty is that once obesity is established, it is hard to reverse. It is believed that once an increased body weight/adiposity content is reached it becomes the “reference” that energy mechanisms adjust towards keeping. Thus, following a weight loss, such as following liposuction/bariatric surgery, the metabolic balance would target this “reference” that represents the previously reached body weight/adiposity content. On the other hand, medical procedures of liposuction and bariatric surgery reduce the level of the adipocytes-produced hormone leptin. This leptin level reduction leads to an increase in food intake and a decrease in energy expenditure. Therefore, the reduced leptin would be among the signals received by the brain to trigger weight regain via processes aiming to re-establish the pre-liposuction/pre-bariatric surgery body weight or adiposity content. We suggest administering leptin so that the brain does not detect the post- liposuction/post-bariatric surgery weight loss; thus, limiting the signals toward weight regain, leading to a better weight control.

## 1. Obesity as a Health Problem

Human energy balance and metabolic homeostasis are among the key processes characterizing life and preserving the integrity of living entities. Both growth and development require energy intake and metabolism. Since energy sources are not always available, humans have the ability to store energy in the form of lipids within the adipose tissue in order to go through hunger periods or food shortage crises. This physiological ability that was, back in time, a survival tool for humans became a burden in modern societies. Indeed, with industrialization and the huge scale of food production, combined with improved economic levels, food availability has significantly increased. This has led to more access to food and, thus, increased food intake. In addition, technology has provided our civilization with facilities and means of accomplishing daily tasks that require reduced human effort [1]. Furthermore, a tendency among populations to engage in less physical activity has developed. All these elements (increased caloric intake and lower exercise rate with a sedentary lifestyle), along with other psychological factors, have resulted in an obesity pandemic.

Obesity is a problem that burdens both health systems and the economy [2,3], as well as the society. During the ongoing coronavirus disease 2019 (COVID-19) crisis [4,5,6], we saw the serious impacts obesity had on COVID-19 severity, as well as the impacts of COVID-19 and its consequences on obesity at a populational level. On one hand, patients suffering from obesity had an increased risk of developing severe forms of COVID-19, as compared to non-obese patients [5]. On the other hand, the measures imposed by governments and health authorities to limit COVID-19, including confinement [7], increased the risk of developing obesity [8], since individuals moved less and had an increased tendency towards food intake [4]. The consumed diet was also of a low quality [8] since the COVID-19 pandemic resulted in an economic crisis [9,10] that impacted the ability of individuals to afford a healthy diet. These interconnections between obesity and COVID-19 put the society within a vicious cycle in which COVID-19 increased obesity development whereas obesity represented a risk factor for severe forms of COVID-19. Furthermore, psychological consequences and mental health issues resulting from COVID-19-related measures worsened the public health profile [11,12].

Obesity prevalence has increased in recent decades, both in adults and in children [13]. It is not only considered a disease [14,15] but also a risk factor or a cause for diverse health problems, such as cardiovascular diseases [16], cancer [17,18,19], sleeping apnea [16], type 2 diabetes [20], dyslipidemia [21,22], and impaired regeneration [23,24], as well as vascular dysfunction, on which obesity has an influence [25]. Obesity pathogenic patterns represent neuroendocrine reprogramming [26] and have even been compared to cancer, in terms of progression, recurrence and metastasis [27], and also to ageing, in terms of molecular pathogenesis and epigenetics [28,29,30]. Different animal models [31,32,33,34] have been developed to study obesity, including molecular and genetic mechanisms [35,36,37,38,39,40], the impact of diets and to test various therapeutic approaches. The animal models, which were generated via diverse approaches, not only allow the study of obesity development but also exploration of the underlying pathways and the pathogenesis and health problems resulting from obesity, or for which the risk factors are increased by obesity. This is of extreme importance, since the key challenge facing the development of efficient molecular therapies against obesity is the limitation in understating the molecular and cellular patterns underlying obesity establishment and development and interindividual variabilities, in terms of obesity progress.

The mathematical vision of obesity is a status that results from having an energy intake (food) higher than energy expenditure (exercise and activity). Therefore, the solution is to reverse this pattern by reducing food intake and/or increasing energy expenditure. Within this context, the most used approaches to manage obesity are both diet control and physical activity [41,42,43]. Dietary intervention is fundamental and essential as the first-line treatment for obese patients, and the main rule of every dietary modification is calorie restriction and consequent weight loss (e.g., [44]). In some cases, pharmacotherapies are also used against obesity [45,46]. In addition, since the diet is not only about caloric density, but also about selected properties of the nutritive elements [42], some additional measures have also been reported in the literature as beneficial for obesity, such as the consumption of tea, coffee [47,48] and curry which include polyphenols [49]. However, when the various methods of managing obesity fail, bariatric surgery or liposuction could represent the last options when obesity has reached a certain level. Such procedures have different impacts on patients since fat distribution between patients is different [50,51,52]. Thus, adiposity-related phenotypes can be different which would justify different approaches regarding patient follow ups.

## 2. Leptin and Weight Regain

Following liposuction or bariatric surgery, there is always a risk of weight regain [53]. This would be explained by the fact that once obesity has been established, the obesity status-related fat content (adiposity) becomes the new biological reference that metabolic balance is centered on [54]. This hypothesis, previously discussed [54] and for which we emphasize that further studies are still required for confirmation, means that energy balance control regulates the metabolism in ways that are assured to keep the fat content as high as the level reached when obesity developed [26]. This includes increased food intake (hunger) and lower energy expenditure. Therefore, preventing weight regain would mean going against biological mechanisms. Such mechanistic metabolic pathways are controlled by various signals and hormones, including those produced by adipose tissue. These regulatory pathways are those that were initially involved in the biological ability to store fat as an energy back up for periods with limited food availability or hunger periods. However, with the development of obesity, this same ability that was meant to be useful is in favor of weight regain following weight loss.

Since bariatric surgery and liposuction result mainly in reduced adipose tissue, one of the key hormonal changes is the decreased levels of the hormones produced by adipose tissue, such as leptin [55]. Leptin, a 160-kDa hormone [56] discovered in 1994 [57], is produced by adipose tissue [58] and acts on regions such as the hippocampus, hypothalamus, and brain stem [59]. In normal physiological conditions, it is a major food intake and energy expenditure regulator [60] that reduces body weight and food intake [61]. It acts to balance lipid storage and to limit the development of fat storage beyond a “normal” level after grown adipose tissue produces leptin that acts to limit food intake, among other effects. Within this context, reduced fat tissue (such as the one occurring following bariatric surgery and liposuction) leads to lower leptin levels, and vice versa. 

Following liposuction or bariatric surgery, the decrease in the circulating leptin would significantly contribute to increase food intake and decrease energy expenditure [55,60], since leptin (which acts against these two effects) is reduced. Thus, the lower leptin levels following weight reduction via liposuction or bariatric surgery contribute to weight regain. This weight regain would aim to restore the adipose tissue to its previous obesity level which had been the new reference for the control of energy balance and adiposity.

Energy homeostasis centers monitor body fat content based on leptin levels, among other signaling hormones and neurotransmitters. These centers switch the metabolism towards storing lipids and increasing adiposity post-liposuction and post-bariatric surgery. Herein, we hypothesize that we could use leptin as a therapeutic agent to limit the weight regain for these patients by “tricking” the energy control centers of the brain. Indeed, providing patients who had bariatric surgeries or liposuction with leptin could be a way of “tricking” the brain since, because by keeping the leptin levels high, the brain would not detect decreased fat storage. In this way, administering leptin post-liposuction or post-bariatric surgery would prevent or limit the triggering of the mechanisms (increased food intake and reduced energy expenditure) that would otherwise aim to restore the adiposity loss.

In obesity there is a resistance to leptin, despite its high circulating levels [62], that represents a characteristic of obesity status [61]. This explains why leptin has not been found to be an efficient anti-obesity therapy [62], due to a state of leptin resistance [61,63]. The leptin resistance mechanism includes the suppression of cytokine signaling 3 and the leptin-stimulated phosphorylation of Tyr(985) on the leptin receptor [64]. With obese patients being insensitive to leptin therapy (exogenous administration of leptin) [62], researchers have tried to investigate potential leptin sensitizers and leptin sensitivity restoration [65,66,67,68] in human and animal studies on leptin administration [69,70].

## 3. Leptin as a Therapeutic Option

**Figure 1:**
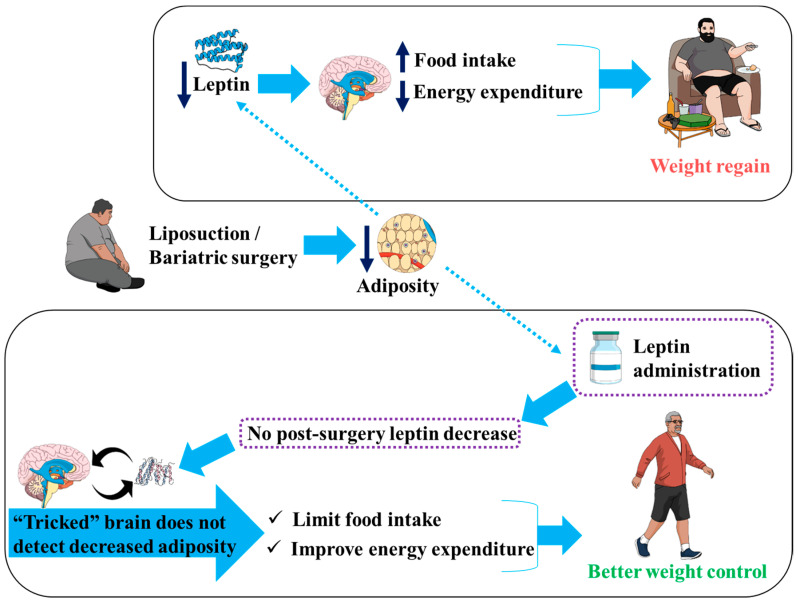
Administering leptin could trick the brain so that it does not detect the post- liposuction/bariatric surgery weight loss and, thus, limit the signals that are toward weight regain.

In our suggested approach, the purpose is not to use the biological properties of leptin and induce an increase in energy expenditure or a decrease in food intake. We rather aim to prevent triggering signals resulting from leptin decrease following the bariatric surgery or the liposuction. Administering leptin after such surgeries would aim to keep leptin levels close to those prior to the surgeries (high) so that the brain areas, with which leptin interreacts to control energy expenditure, food intake and thermogenesis [59], do not detect leptin decrease. Therefore, the brain would be “blind” to the adiposity decrease (Figure 1), which would prevent weight regain due to leptin decrease that follows liposuction or bariatric surgery.

Leptin could be an important addition to the therapies prescribed after bariatric surgeries or liposuction. As pharmacovigilance could be against giving high dosages of leptin for long periods, we suggest proceeding similarly to drug addiction detoxification therapies or nicotine patches. First give leptin so as to have in vivo levels similar to those prior to the bariatric surgery or liposuction and, after that, gradually decrease the injected amount of leptin over a period of time until eventually leptin administration ceases. This way would limit the detection of a sudden leptin decrease and thus prevent triggering strong signals towards weight regain. Such approaches would also facilitate and allow a potential biological adaptation to the new metabolic phenotype with lower adiposity. The final purpose would be to use leptin administration (gradually decreasing) during the period of time required by the body in order to adapt to the changes in leptin levels from obesity to post-bariatric surgeries or liposuction, via avoiding the detection by the brain of the sudden decrease in leptin levels. The leptin doses to administer and the leptin therapy duration are yet to be optimized. It is worth exploring in diverse contexts, including animal trials and clinical studies, and even with a focus on the neurological interactions, since leptin resistance and energy balance mechanisms could involve neuroplasticity. Of course, combining such leptin therapy to a healthy lifestyle, including a balanced diet, physical activity and psychological well-being, would lead to better long-term outcomes.

## 4. Perspectives

We believe that the theory we have introduced via this piece of writing could represent a starting point in the context of leptin-based therapies and not only for obesity. The non-weight related effects of leptin, including immune response modulation, inflammation [71,72], hematopoiesis [73] and metabolism-immune system interplay [74] could all be therapeutically targeted. Moreover, although liposuction and bariatric surgery are not the only approaches associated with the weight regain problem, we focused on liposuction and bariatric surgery because patients who have liposuction and bariatric surgery are usually those with the most severe forms of obesity and, therefore, with whom the weight regain problem would be most noticeable.

## Data Availability

Not applicable.
